# Integrating the gut microbiome into therapeutic drug monitoring and model-informed precision dosing: a clinical pharmacy perspective

**DOI:** 10.3389/fphar.2026.1938639

**Published:** 2026-10-19

**Authors:** Ting Zhang, Shiqian Wang, Xiafang Xu, Renjie Xu

**Affiliations:** Department of Pharmacy, Shaoxing Maternity and Child Health Care Hospital, Shaoxing, China

**Keywords:** clinical pharmacy, gut microbiome, interindividual pharmacokinetic variability, model-informed precision dosing, pharmacomicrobiomics, therapeutic drug monitoring

## Abstract

Interindividual variability in drug response remains a central obstacle to effective pharmacotherapy, and much of it persists after accounting for the pharmacogenomic factors that currently guide individualized treatment. Therapeutic drug monitoring (TDM) and model-informed precision dosing (MIPD) have become a mature, covariate-driven discipline that tailors dose to renal function, body size, age, and genotype, yet one determinant of drug exposure is conspicuously absent from these frameworks: the gut microbiome. Although gut bacteria are now known to alter the pharmacokinetics of many drugs, microbial variation is not represented in dosing models and is instead absorbed into unexplained residual variability. This review reframes the gut microbiome as a measurable, dosing-relevant covariate rather than a mechanistic phenomenon. We organize the mechanisms by which gut bacteria shape drug pharmacokinetics into four classes (biotransformation, inactivation, bioaccumulation, and indirect modulation of host enzymes and enterohepatic recirculation) and summarize the landmark evidence that microbial variation produces measurable interindividual differences in exposure and response, graded explicitly by evidential tier so that causal and associative findings are not conflated. We then locate the microbiome blind spot within current TDM and MIPD, and propose an operational framework specifying what microbial features to measure, how they should be sampled and measured reproducibly, and how to integrate them into population-pharmacokinetic, Bayesian, machine-learning, and physiologically based dosing models, setting out for each route its data requirements, validation strategy, limitations, and current prototyping status, and illustrating the framework with three worked examples of differing evidential maturity. Finally, we translate the framework into strategies actionable by clinical pharmacists and delineate the biological, methodological, and translational obstacles ahead, including the workflow burden, cost, and biomarker-qualification requirements that adoption would entail. By treating the microbiome as a covariate, this perspective charts a route toward microbiome-informed precision dosing at the bedside.

## 1 Introduction

Interindividual variability in drug response is a defining challenge of clinical pharmacotherapy. Patients given identical doses of the same drug often reach very different systemic exposures and clinical outcomes. These range from therapeutic failure to dose-limiting toxicity, with costs in adverse drug reactions, prolonged admissions, and delayed recovery. Therapeutic drug monitoring (TDM) and, more recently, model-informed precision dosing (MIPD) were developed to close this gap. Both individualize dose from measured drug concentrations and patient-specific covariates (Darwich et al., 2021). Even so, much of the variability these models seek to explain still escapes the covariates used in routine care (Sharma et al., 2019).

For 2 decades, pharmacogenomics has been the main lens on unexplained variability. It links polymorphisms in drug-metabolizing enzymes and transporters to differences in exposure and effect (Hassan et al., 2021). This framework is now embedded in precision-medicine practice. Host genetics alone, however, accounts for only a fraction of observed interpatient differences, leaving a persistent explanatory deficit (Spanogiannopoulos et al., 2016).

Attention has therefore turned to the gut microbiome. The trillions of microorganisms that colonize the human intestine encode a metabolic repertoire that far exceeds that of the host. This “second genome” is an under-appreciated determinant of xenobiotic metabolism (Human Microbiome Project Consortium, 2012; Spanogiannopoulos et al., 2016). The resulting field of pharmacomicrobiomics examines how variation in microbial composition and function shapes drug absorption, disposition, and response (ElRakaiby et al., 2014; Saad et al., 2012).

When this lens is narrowed to pharmacokinetics, the evidence is striking. Gut bacteria alter drug fate through biotransformation, inactivation, intracellular bioaccumulation, and modulation of host enzymes and transporters. Their clinical relevance was established decades ago. Inactivation of digoxin by intestinal bacteria was shown to lower serum concentrations and to reverse upon antibiotic exposure (Lindenbaum et al., 1981). Contemporary work has scaled this observation into systematic maps. A survey of 76 gut bacterial strains against 271 oral drugs found that two-thirds are chemically modified by at least one strain, and the responsible genes were identified (Zimmermann et al., 2019a). Gnotobiotic and modeling approaches have since separated host from microbial contributions to drug pharmacokinetics and toxicity in quantitative terms (Zimmermann et al., 2019b). The chemical logic of these reactions is now broadly catalogued (Koppel et al., 2017), and their aggregate effect on drug disposition and pharmacokinetic-pharmacodynamic variability is increasingly documented in humans (Tsunoda et al., 2021).

In parallel, TDM and MIPD have matured into sophisticated dosing frameworks. Bayesian, AUC-guided monitoring is now recommended over trough-based dosing for vancomycin (Rybak et al., 2020). Structured MIPD approaches are advocated for antibiotics (Wicha et al., 2021), and international guidance endorses concentration-guided individualization across drug classes in the critically ill (Abdul-Aziz et al., 2020). These frameworks incorporate renal function, body size, age, and increasingly pharmacogenomic data, yet they omit the microbiome. The gut microbial contribution to drug exposure is, in effect, an unmodeled covariate, a blind spot at the centre of precision dosing.

Three bodies of prior work bound the contribution of this review, and it is worth stating what each has and has not established. Mechanistic pharmacomicrobiomics reviews have catalogued drug-microbiome interactions and their clinical associations comprehensively (Doestzada et al., 2018; Weersma et al., 2020; Zhao et al., 2023; Zimmermann et al., 2021). They are organized by biology and not by dosing decision, and they stop short of specifying how a microbial measurement would enter a dose calculation. Tsunoda et al. (2021) reviewed the microbiome’s contribution to disposition and pharmacokinetic-pharmacodynamic variability from within clinical pharmacology, framing the problem in pharmacokinetic language. That review still treated the microbiome as a source of variability to be recognized, not a covariate to be measured and modeled. Most directly, Zimmermann-Kogadeeva et al. (2020), building on Zimmermann et al. (2019b), developed physiologically based models that represent microbial drug-metabolizing activity explicitly and predict its effect on systemic exposure. This is the closest prior art, and it establishes the decisive feasibility point that microbial metabolism can be represented quantitatively inside a pharmacokinetic model. It did so, however, in gnotobiotic mice differing in a single microbiome-encoded enzyme, for mechanistic attribution. It did not address which microbial feature a clinician could measure, how that measurement would be standardized, how it would propagate into the Bayesian and population-model machinery that governs dosing in practice, or what a pharmacist would do differently as a result.

What this review adds is therefore not new mechanism but a translational specification, in four parts. First, we organize microbial effects on pharmacokinetics into four operational classes, including intracellular bioaccumulation, which post-dates most earlier taxonomies and carries distinct modeling consequences because it alters free luminal drug without generating a metabolite. Second, we recast the problem in TDM and MIPD terms, identifying the microbiome as an unmodeled covariate within an architecture already built to absorb covariates. Third, we specify four modeling routes by which a microbial measurement can reach a dose, with their data requirements, validation strategies, limitations, and prototyping status (Table 1). Fourth, we translate the framework into actions available to pharmacists now, and state the evidentiary, economic, and regulatory conditions for adopting its quantitative form. The gap addressed is thus a specification gap rather than a discovery gap: the biology is established and the modeling demonstrably feasible in principle, yet no published framework connects a measurable microbial feature to a dose adjustment through the workflows clinicians actually use.

**TABLE 1:** The four covariate routes: data requirements, validation strategy, limitations, and prototyping status.

Route	Where the covariate acts	Data required	Validation strategy	Principal limitations	Prototyped?
1. Covariate on a structural PK parameter (popPK)	Bioavailability (F) for gut-metabolized drugs; apparent clearance or reabsorption for enterohepatically recirculated drugs	Paired biomarker + concentration-time data in a cohort powered for covariate estimation; principal non-microbial determinants measured as competing covariates	Goodness-of-fit and parameter precision → predictive checks → prospective demonstration of improved individual exposure prediction vs. the same model without the microbial term; pre-specified functional form; external cohort (internal cross-validation insufficient)	Largest patient numbers; selection bias and overstated effect size in small datasets (Ribbing and Jonsson, 2004); drug-specific and non-transferable; covariate estimated observationally so inherits §5.1 confounding; presupposes biomarker stability	No. No published prototype with a microbial covariate
2. Metabotype-informed Bayesian prior	Selection or weighting among competing population priors	A discrete, reproducible metabotype classification + ≥2 population models (or one with subgroup-specific parameters)	Compare predictive performance (bias, imprecision in predicted concentrations) of metabotype-selected vs. pooled prior in patients not used to derive the classification	Discretizing a continuous gradient discards information and is threshold-sensitive; a misclassified patient may fare worse than under a pooled prior; quantifies nothing mechanistically	No for a microbial metabotype, but prior selection/averaging is established in vancomycin dosing (Broeker et al., 2019), supplying the template. Lowest-cost first prototype (see §5.3)
3. Microbiome features in an ML predictor	Direct prediction of exposure or dose from high-dimensional features	Steepest requirement: many patients relative to feature count; feature selection nested inside cross-validation; external cohort indispensable	Nested cross-validation → external validation → interpretability assessment, since an unexplainable recommendation is unlikely to be acted on (Swartling et al., 2025)	Overfitting risk; predictor opacity; may exploit confounded signal (diet, co-medication). Currently rests on two studies from adjacent domains: tacrolimus ML with conventional covariates (Woillard et al., 2021) and microbiome-plus-host prediction of glycemic, not drug-exposure, response (Zeevi et al., 2015)	No. No published prototype predicting drug exposure from microbiome features
4. Mechanistic intestinal compartment (PBPK)	Explicit enzymatic activity in an intestinal compartment	Mechanistic parameters rather than a cohort: microbial enzyme specific activity, size and regional distribution of the metabolizing population, transit kinetics, drug and metabolite absorption	Parameter plausibility → prediction of observed concentration-time profiles → ideally prediction of a deliberate perturbation (e.g., antibiotic exposure)	Many parameters requiring independent determination; high sensitivity to the least well-characterized (regional microbial load, transit); no human parameterization	Yes, partially: microbial activity represented explicitly and used to predict systemic exposure with quantitative ΔAUC attribution — in gnotobiotic mice only (Zimmermann et al., 2019b; Zimmermann-Kogadeeva et al., 2020)

ML, machine learning; PBPK, physiologically based pharmacokinetic; PK, pharmacokinetic; popPK, population pharmacokinetic; ΔAUC, germ-free-to-colonized difference in area under the concentration-time curve. Routes are ordered from empirical to mechanistic and are complementary rather than mutually exclusive. The prototyping column answers the question directly: three of the four routes have no published prototype of any kind for a microbial covariate, and the fourth has been prototyped only in gnotobiotic mice.

This review addresses that gap as follows. We first synthesize the mechanisms by which the gut microbiome shapes drug pharmacokinetics, and the landmark evidence that these mechanisms drive measurable interindividual variability, graded by evidential tier. We then examine the current state of TDM and MIPD and locate the microbiome blind spot within it. Building on this, we propose a framework for integrating the microbiome as a measurable covariate into population-pharmacokinetic, Bayesian, machine-learning, and physiologically based dosing models. We then translate this framework into practical strategies for clinical pharmacists: covariate selection, biomarker measurement, antibiotic-drug sequencing, and patient stratification. By reframing the microbiome from a mechanistic curiosity into a dosing-relevant variable, we aim to move the field from recognizing that microbes affect drugs toward microbiome-informed precision dosing at the bedside.

## 2 Mechanisms: how the gut microbiome shapes drug pharmacokinetics

The gut microbiome is a metabolically active organ. It acts on orally administered drugs through a small, recurring set of biochemical strategies rather than an open-ended catalogue of reactions (Spanogiannopoulos et al., 2016). Its chemistry is largely reductive and hydrolytic, complementing the oxidative and conjugative metabolism of the host. It operates at high density in the distal intestine, where many drugs and their excreted metabolites accumulate (Collins and Patterson, 2020). Four mechanistic classes account for most documented effects on pharmacokinetics: direct biotransformation of the parent drug, inactivation to less active or toxic species, non-metabolic bioaccumulation, and indirect modulation of host drug-metabolizing enzymes, transporters, and enterohepatic recirculation (Figure 1). These classes are not mutually exclusive for a given drug. Distinguishing them, however, clarifies where microbial variation can enter, and ultimately be modeled within, a pharmacokinetic framework.

**FIGURE 1:**
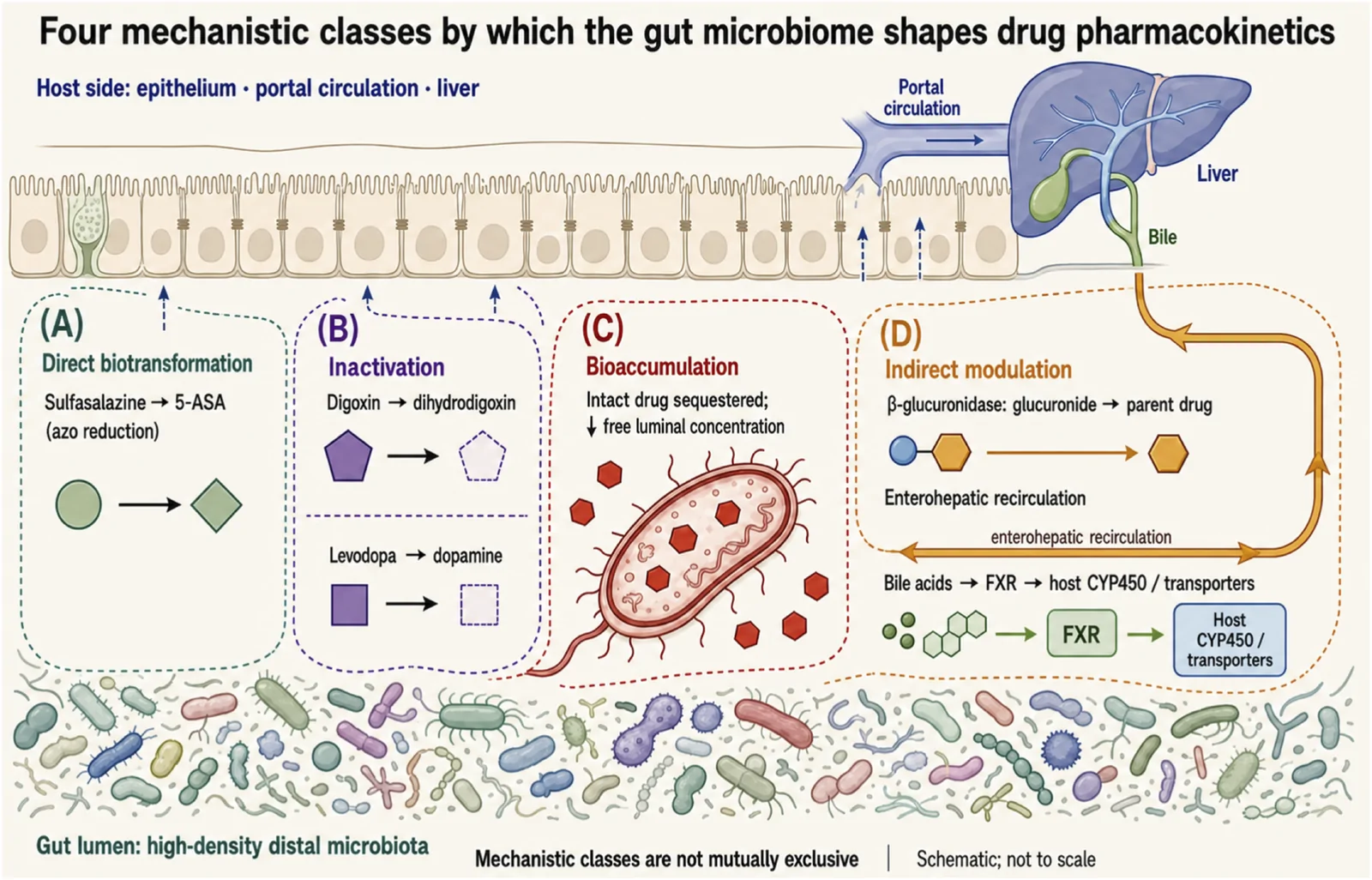
Four mechanistic classes by which the gut microbiome shapes drug pharmacokinetics. Within a single intestinal setting, gut bacteria act on orally administered drugs through four broad and non-mutually-exclusive routes. **(A)** Direct biotransformation of the parent compound by reductive and hydrolytic chemistry, for example, azoreductive activation of the prodrug sulfasalazine to 5-aminosalicylate. **(B)** Inactivation to less active or toxic species, illustrated by reduction of digoxin by *Eggerthella lenta* and decarboxylation of levodopa by bacterial tyrosine decarboxylase. **(C)** Intracellular bioaccumulation of intact drug without chemical modification, which lowers the free luminal concentration available for absorption. **(D)** Indirect modulation of host disposition, in which bacterial β-glucuronidases deconjugate glucuronides to prolong enterohepatic recirculation, and microbial bile-acid metabolism modulates farnesoid X receptor signaling and host cytochrome P450 and drug-transporter expression. Reductive and hydrolytic reactions predominate and occur at highest density in the distal intestine. The four classes are not mutually exclusive for a given drug.

Direct biotransformation covers the reactions by which gut bacteria convert a drug into structurally distinct metabolites. Reductive and hydrolytic transformations dominate, and their scope is now defined at community scale. Screening 76 gut bacterial strains against 271 orally administered drugs showed that 176 of the drugs, about two-thirds, are chemically modified by at least one strain, and the responsible bacterial gene products were identified in many cases (Zimmermann et al., 2019a). Several of these reactions are therapeutically intended. Azo reduction cleaves the prodrug sulfasalazine to release active 5-aminosalicylate in the colon, at a rate that varies between individuals with the resident community (Sousa et al., 2014). The same chemistry classically activates prontosil to sulfanilamide (Wilson and Nicholson, 2017). Bacterial arylamine N-acetyltransferases further acetylate 5-aminosalicylate, showing that microbes contribute conjugative as well as reductive chemistry (Deloménie et al., 2001). Direct biotransformation is not confined to prodrug activation, and it reaches drugs managed by TDM today. Gut commensals, including *Faecalibacterium prausnitzii*, convert the immunosuppressant tacrolimus to a less potent C-9 keto-reduced metabolite in anaerobic culture, supplying a candidate mechanism for the cohort-level association between this taxon and tacrolimus dose requirement discussed in Section 3 (Guo et al., 2019). This matters here because tacrolimus is a narrow-therapeutic-index agent already under routine concentration monitoring. A microbial contribution to its disposition would therefore enter an existing dosing workflow instead of requiring a new one. The gut thus operates as a distinct site of biotransformation, whose output depends on the enzymatic activities present (Sousa et al., 2008).

A second class of reactions inactivates drugs by turning an active molecule into an inert or harmful product, with direct pharmacodynamic and safety consequences. The cardiac glycoside digoxin is reduced to inactive dihydrodigoxin by strains of *Eggerthella lenta* that carry a cardiac glycoside reductase operon, so patients harboring reducing strains reach lower serum concentrations (Haiser et al., 2013). Levodopa is decarboxylated to dopamine by bacterial tyrosine decarboxylases in the proximal small intestine. This restricts the fraction available for absorption and correlates with the dose required to control Parkinsonian symptoms (van Kessel et al., 2019). Microbial inactivation can also generate toxicity. Gut bacterial conversion of the antiviral sorivudine yields bromovinyluracil, which irreversibly inhibits host dihydropyrimidine dehydrogenase and precipitates lethal accumulation of co-administered 5-fluorouracil (Okuda et al., 1997). This chain of events was implicated in eighteen patient deaths in Japan within 40 days of the drug’s approval (Okuda et al., 1998). Bacterial nitroreduction supplies a further example of microbially generated harm. Reduction of nitrazepam to 7-aminonitrazepam by intestinal bacteria is required for the drug’s teratogenic effect in rats, and the effect is attenuated when the gut flora is suppressed (Takeno and Sakai, 1991). The logic parallels sorivudine. A microbial reaction, not the parent drug, generates the species responsible for the toxicity. The relevant interindividual variable is therefore microbial enzymatic capacity, not host exposure to the administered compound.

Bioaccumulation is a distinct route in which bacteria sequester a drug intracellularly without chemically transforming it. Systematic screening of fifteen structurally diverse drugs against twenty-five gut bacterial strains found that more than half of the newly identified interactions reflected storage of the intact drug inside the cell, often without affecting bacterial growth (Klünemann et al., 2021). The antidepressant duloxetine is the best-characterized case. It binds several bacterial metabolic enzymes and shifts the metabolite secretion of accumulating strains. In a defined community of accumulators and non-accumulators it markedly alters composition through metabolic cross-feeding, and bioaccumulating bacteria attenuate the behavioural response of *Caenorhabditis elegans* to the drug (Klünemann et al., 2021). Duloxetine thus illustrates both halves of the relationship in one system: the community changes the drug’s availability, and the drug reshapes the community that determines it. The sequestered drug is neither degraded nor immediately available, so this process alters the free concentration presented for absorption and can reshape the surrounding microbial community. It has consequently been recognized as a third category of microbiome-drug interaction, alongside biotransformation and inactivation (Cohen and Kelly, 2022). For modeling purposes bioaccumulation is distinctive, and awkward. No metabolite is produced, so the interaction is invisible to metabolite-based assays and must be inferred from parent-drug disappearance or from direct intracellular measurement.

Beyond acting on the drug itself, the microbiome reshapes host drug handling and enterohepatic recirculation, extending its reach to systemically administered agents. Bacterial β-glucuronidases hydrolyze the glucuronide conjugates that the host has marked for excretion, regenerating the parent compound in the gut lumen for reabsorption. This reactivation prolongs enterohepatic recirculation and drives toxicity. It is seen when the inactive glucuronide of the irinotecan metabolite SN-38 is deconjugated to its active form in the intestine (Wallace et al., 2010), and the same enzymes reactivate glucuronidated estrogens as part of the estrobolome (Ervin et al., 2019). The structural diversity of these enzymes across the microbiome has been catalogued in detail (Pollet et al., 2017). The same chemistry applies to mycophenolic acid, whose glucuronide is deconjugated by gut bacterial β-glucuronidase. Eliminating this activity with oral vancomycin relieves mycophenolate-induced gastrointestinal toxicity in mice (Taylor et al., 2019), and in humans co-administration of trimethoprim-sulfamethoxazole perturbs the community and lowers systemic mycophenolic acid exposure (Dukaew et al., 2023). Mycophenolic acid is thus the one agent in this class for which mechanistic, interventional-animal, and prospective human pharmacokinetic evidence are all available, which is why it recurs as a worked example in Section 5.4.3.

Microbial metabolism of bile acids modulates farnesoid X receptor signaling, an axis that governs the expression of host transporters and metabolic enzymes (Wahlström et al., 2016). The reach of this indirect control extends to systemic phase I metabolism. Germ-free mice show markedly lower hepatic CYP3A activity than specific-pathogen-free controls, approximately fourfold higher plasma exposure to the CYP3A substrate midazolam after intraperitoneal administration, and an approximately fourteenfold higher brain concentration at 180 min (Togao et al., 2020). Because the drug was given parenterally, the effect cannot be attributed to luminal metabolism and must reflect microbial regulation of host enzyme expression. That makes this the clearest quantified instance of indirect modulation, consistent with broad microbial regulation of host phase I and phase II enzymes and drug transporters (Klaassen and Cui, 2015).

Taken together, these four mechanistic classes give a reasonably complete vocabulary for how gut bacteria alter drug disposition. Translational value now depends less on discovering more reactions than on determining which of them matter, in which patients, and to what quantitative degree. The central obstacle is that host and microbial processes often perform overlapping transformations, so an observed metabolite cannot be assigned to either source without controlled measurement. Combining gnotobiotic mouse models with bacterial genetics has begun to resolve this, separating host from microbial contributions to drug pharmacokinetics and toxicity in quantitative terms (Zimmermann et al., 2019b). Embedding these measurements within physiologically based pharmacokinetic models then offers a route to predicting systemic exposure as a function of microbial activity (Zimmermann-Kogadeeva et al., 2020). Whether such mechanistic quantification translates into measurable interindividual variability in real patients is the question taken up next.

## 3 Evidence for microbiome-driven interindividual pharmacokinetic variability

Establishing that gut bacteria can transform drugs does not by itself prove that microbial variation produces clinically meaningful differences between patients. The relevant question for precision dosing is whether interpersonal differences in microbial composition and enzymatic capacity translate into measurable, and occasionally dominant, differences in drug exposure and response (Spanogiannopoulos et al., 2016). The strength of this evidence is uneven, and a dosing covariate must ultimately rest on causal, not correlative, support. We therefore grade it explicitly here and in Table 2 using four tiers. Tier 1 is causal and controlled evidence: germ-free or gnotobiotic animals, defined strains, or microbiota transfer. Tier 2 is prospective human interventional or controlled co-administration data. Tier 3 is human observational or cohort association. Tier 4 is *ex vivo* human microbiota, *in vitro*, or invertebrate-model evidence. The tiers rank not scientific quality but proximity to the inference a dosing model requires, namely, that changing the microbial feature changes exposure. Table 2 summarizes the landmark drug-microbiome interactions and their documented pharmacokinetic consequences.

**TABLE 2:** Landmark drug-microbiome interactions and their pharmacokinetic consequences.

Drug	Responsible microbe/ enzyme	Reaction type	Pharmacokinetic/ clinical consequence	Evidence tier	Evidence
Sulfasalazine	Bacterial azoreductase	Azo reduction (prodrug activation)	Individual-dependent release of active 5-ASA	Tier 4 (*ex vivo*/ *in vitro* human microbiota)	Sousa et al. (2014)
Prontosil	Bacterial azoreductase	Azo reduction (prodrug activation)	Historical paradigm case of microbial prodrug activation to sulfanilamide; no modern quantified PK data	Tier 4 (historical/ mechanistic)	Wilson and Nicholson (2017)
Digoxin	*Eggerthella lenta* (cardiac glycoside reductase, *cgr*)	Reduction (inactivation)	↓ serum concentration; excretor vs. non-excretor phenotype	Tier 3 (human cohort) + Tier 1 (defined strains, mice)	Lindenbaum et al. (1981); Haiser et al. (2013)
Levodopa	Bacterial tyrosine decarboxylase (interspecies pathway)	Decarboxylation (inactivation)	↓ absorbable fraction; ↑ dose requirement	Tier 3 (human cohort) + Tier 1 (defined pathway, mice)	van Kessel et al. (2019); Maini Rekdal et al. (2019)
Mesalazine (5-ASA)	Bacterial arylamine N-acetyltransferases	Acetylation (inactivation)	↓ clinical efficacy in inflammatory bowel disease	Tier 3 (human cohort)	Mehta et al. (2023)
Sorivudine (with 5-fluorouracil)	Gut bacteria (reductive conversion to bromovinyluracil)	Reduction → host DPD inhibition	Lethal 5-fluorouracil accumulation	Tier 3 (human case series, 18 deaths; Okuda et al., 1998) + Tier 1 (animal mechanism; Okuda et al., 1997)	Okuda et al. (1997), Okuda et al. (1998)
Nitrazepam	Bacterial nitroreductase	Nitroreduction to 7-aminonitrazepam (toxic metabolite)	Microbially generated metabolite required for teratogenicity; effect attenuated by flora suppression	Tier 1 (rats, flora suppression)	Takeno and Sakai (1991)
Tacrolimus (biotransformation)	Gut commensals incl. *Faecalibacterium prausnitzii*	C-9 keto reduction (inactivation)	Conversion to less potent metabolite; candidate mechanism for the cohort dose association below	Tier 4 (anaerobic culture)	Guo et al. (2019)
Duloxetine	Multiple gut bacterial strains (no transformation)	Intracellular bioaccumulation	↓ free luminal drug; drug reshapes community via cross-feeding; attenuated drug response in *C. elegans*	Tier 4 (*in vitro* + invertebrate)	Klünemann et al. (2021)
Irinotecan (SN-38)	Bacterial β-glucuronidase	Deconjugation-reactivation	Prolonged enterohepatic recirculation; ↑ gastrointestinal toxicity; high-turnover metabotype	Tier 1 (enzyme inhibition, mice) + Tier 4 (human metabotype)	Wallace et al. (2010); Guthrie et al. (2017)
Mycophenolic acid	Bacterial β-glucuronidase	Deconjugation-reactivation	↓ systemic exposure on TMP-SMX co-administration (human); relief of GI toxicity when activity eliminated (mouse)	Tier 2 (prospective human co-administration) + Tier 1 (mouse intervention)	Dukaew et al. (2023); Taylor et al. (2019)
Midazolam	Microbial regulation of host hepatic CYP3A	Indirect (host phase I enzyme modulation)	≈4-fold ↑ plasma AUC and ≈14-fold ↑ brain concentration in germ-free vs. SPF mice after IP dosing	Tier 1 (germ-free vs. SPF mice)	Togao et al. (2020)
Tacrolimus (cohort association)	*Faecalibacterium prausnitzii* (association)	Microbial correlate	↑ dose requirement (narrow therapeutic index)	Tier 3 (human cohort)	Lee et al. (2015)
Simvastatin	Microbial secondary bile acids	Indirect (pharmacodynamic conditioning)	Baseline bile acids predict magnitude of LDL-cholesterol lowering	Tier 3 (human cohort)	Kaddurah-Daouk et al. (2011)
Metformin	Gut microbiome (causal; FMT-transferable)	Indirect	Therapeutic effect partly microbiome-mediated	Tier 1 (microbiota transfer to germ-free mice) + Tier 2 (human treatment)	Wu et al. (2017)
Fluoropyrimidines (5-FU, floxuridine)	Defined bacterial genes (host-microbe co-metabolism)	Indirect (bidirectional modulation of drug efficacy)	Bacterial genotype tunes chemotherapeutic efficacy in either direction; invertebrate host, no mammalian PK data	Tier 4 (*C. elegans*)	García-González et al. (2017)

5-ASA, 5-aminosalicylate; AUC, area under the concentration-time curve; *cgr*, cardiac glycoside reductase; CYP3A, cytochrome P450 3A; DPD, dihydropyrimidine dehydrogenase; FMT, fecal microbiota transplantation; GI, gastrointestinal; IP, intraperitoneal; PK, pharmacokinetic; SN-38, active metabolite of irinotecan; SPF, specific-pathogen-free; TMP-SMX, trimethoprim-sulfamethoxazole. Species names are italicized. Rows are ordered by mechanistic class (biotransformation, inactivation, bioaccumulation, indirect modulation). Evidence tiers: Tier 1, causal/controlled (germ-free, gnotobiotic, defined strains, microbiota transfer); Tier 2, prospective human interventional or controlled co-administration; Tier 3, human observational/cohort association; Tier 4, *ex vivo* human microbiota, *in vitro*, or invertebrate model. Where two tiers are listed, independent lines of evidence at both levels exist. Model species are stated explicitly wherever the evidence is non-human.

Table 2 is restricted to exogenous, systemically or enterally administered drugs for which a specific microbial mechanism and a documented pharmacokinetic or directly dose-relevant clinical consequence have both been reported. It therefore excludes endogenous substrates (for example, the estrobolome reactivation of glucuronidated estrogens, mechanistically identical to the SN-38 row and discussed in Section 2), microbiome-mediated effects without a characterized pharmacokinetic consequence, and drugs whose only reported interaction is a change in community composition. Rows are grouped by mechanistic class. The evidence tier follows the four-tier scheme defined in Section 3 and denotes proximity to the causal inference a dosing model requires, not scientific quality.

### 3.1 Tier 4 and Tier 3 — reproducible metabolizer phenotypes

Individuals carry reproducible microbial metabolic capacities that sort them into discrete metabolizer phenotypes, mirroring the language of pharmacogenetics. The earliest quantitative example remains digoxin, which is reduced to inactive metabolites in roughly one in ten treated patients, cleanly separating “excretors” from “non-excretors” by their resident flora (Lindenbaum et al., 1981). *Ex vivo* screening of personal microbiota against panels of drugs has since generalized this pattern. The extent of metabolism for many compounds is specific to the individual donor and reproducible on repeat sampling (Javdan et al., 2020). For irinotecan, individuals whose communities show high β-glucuronidase turnover form a distinct metabotype, predicted to carry elevated risk of drug reactivation and gastrointestinal toxicity; this links a measurable microbial activity to a stratifiable clinical phenotype (Guthrie et al., 2017). These are Tier 4 findings in the sense that matters here: the phenotype is demonstrated on human material outside the body, establishing stable interindividual capacity without showing that the capacity governs exposure in the donor patient.

### 3.2 Tier 3 — microbial features tracking exposure and dose in patient cohorts

In patient cohorts, particular microbial features track drug exposure and the dose actually required to reach target. Among kidney transplant recipients, the fecal abundance of *Faecalibacterium prausnitzii* in the first week after transplantation correlates with subsequent tacrolimus dose escalation, identifying a microbial correlate of a narrow-therapeutic-index immunosuppressant (Lee et al., 2015). The *in vitro* conversion of tacrolimus by this taxon now offers a mechanistic candidate for that association (Guo et al., 2019). In Parkinson’s disease, the abundance of bacterial tyrosine decarboxylase genes correlates with the levodopa dose needed to control symptoms, connecting a specific microbial enzyme to a dosing endpoint (van Kessel et al., 2019). The interspecies bacterial pathway responsible for this decarboxylation has been defined mechanistically (Maini Rekdal et al., 2019). **Tier 2** Prospective human co-administration data extend the pattern to drug-drug-microbiome interactions: trimethoprim-sulfamethoxazole alters the gut community and reduces systemic exposure to mycophenolic acid, a change attributable to shifts in microbial reactivation of the drug (Dukaew et al., 2023). This last study is, to our knowledge, the closest existing approximation to the evidence a dosing covariate would need, because a deliberate perturbation of the microbiome was followed by measured change in human systemic exposure of a monitored drug.

Microbial variation shapes not only drug concentration but therapeutic effect, closing the loop between exposure and outcome. Baseline concentrations of bacterially derived secondary bile acids predict the magnitude of LDL-cholesterol lowering achieved with simvastatin, indicating that microbial metabolism conditions the pharmacodynamic response (Kaddurah-Daouk et al., 2011) (Tier 3). In inflammatory bowel disease, microbial acetyltransferases inactivate 5-aminosalicylate, and the extent of this metabolism corresponds to reduced clinical efficacy, providing a direct human link between a microbial reaction and treatment success (Mehta et al., 2023) (Tier 3). The therapeutic action of metformin is itself partly mediated by the gut microbiome. Transfer of metformin-altered microbiota to germ-free recipients improves glucose tolerance, showing that microbial contributions can be causal rather than merely correlative (Wu et al., 2017) (Tier 1 for causality in mice, alongside Tier 2 human treatment data).

### 3.3 Tier 1

Controlled experimental systems now attribute variability to the microbiota quantitatively and map it onto bacterial genotype, supplying the causal grounding that human association studies lack. Host and microbial pathways frequently perform the same transformation, so the microbial contribution cannot be read from observation alone. Combining gnotobiotic mice with defined bacterial strains resolves this overlap. It separates host from microbial contributions to a shared transformation, and attributes a defined, measurable fraction of the germ-free-to-colonized difference in exposure (the ΔAUC) to the microbiota (Zimmermann et al., 2019b). That attribution was demonstrated for brivudine, using mice differing in a single microbiome-encoded enzyme, and extended to clonazepam as a drug subject to multiple competing metabolic routes. The microbial fraction is therefore drug-dependent, and its magnitude has been quantified for these agents but not for the clinical examples discussed above. For digoxin and levodopa, the case that microbial status can dominate exposure rests instead on the human and strain-level evidence already cited (Haiser et al., 2013; Lindenbaum et al., 1981; van Kessel et al., 2019). It remains an inference from metabolizer-phenotype data, not a directly partitioned ΔAUC. In *Caenorhabditis elegans*, host-microbe co-metabolism determines the efficacy of fluoropyrimidine chemotherapy, with defined bacterial genes tuning the response in either direction (García-González et al., 2017) (Tier 4: an invertebrate host, included because it demonstrates genetically defined bidirectional control, not because it supports extrapolation to human exposure). Genetic tools for *Eggerthella lenta* further connect strain-level genomic variation to drug-metabolizing phenotype, so the presence or absence of specific gene variants predicts metabolic activity (Bisanz et al., 2020). These controlled systems establish causality. Quantitative extrapolation from model organisms and gnotobiotic animals to dosing in patients, however, still requires prospective human validation.

Collectively, these findings establish microbial status as a measurable, and in specific cases dominant, source of interindividual variability in both drug exposure and response. This satisfies the first requirement for treating the microbiome as a dosing-relevant variable. The contribution of the microbiome to drug disposition and pharmacokinetic-pharmacodynamic variability is increasingly acknowledged within clinical pharmacology (Tsunoda et al., 2021). What the current evidence largely lacks is the prospective, quantitative, and reproducible character required of a dosing covariate. As Table 2 makes explicit, the causal evidence is concentrated in Tier 1 animal systems, whereas the drugs of greatest clinical interest are supported mainly by Tier 3 human association. Several agents pair Tier 1 causal evidence with Tier 3 human association. Only mycophenolic acid couples Tier 1 causality to Tier 2 prospective human exposure data, which is the combination a dosing covariate ultimately requires. Most human data are associative and retrospective, effect sizes are rarely expressed in units a pharmacokinetic model could use, and standardized measurement of the responsible microbial features is uncommon.

One further limitation must be raised here rather than deferred, because it conditions how the framework of Section 5 should be read: confounding. Microbial signatures are entangled with the disease being treated and with concomitant medication. The gut microbiome signature of type 2 diabetes, for instance, could be resolved only after controlling for antidiabetic drug exposure (Forslund et al., 2015). A large share of between-person microbiome variation is explained by diet, physiology, stool consistency, and medication use (Falony et al., 2016; Zhernakova et al., 2016). Several Tier 3 associations above are therefore vulnerable: an organism or gene tracking dose requirement may mark disease severity, diet, or co-medication, not drug-metabolizing capacity. Any candidate biomarker in Section 5 inherits this structured confounding. Neither the measurement layer of Section 5.2 nor the modeling routes of Section 5.3 can be evaluated as if the microbial feature were an independent patient characteristic on the footing of body weight or creatinine clearance. The framework that follows should thus be read as a proposal conditioned on these problems being handled, not one that assumes them away. This evidentiary gap, rather than any doubt about biological reality, explains why the mature dosing frameworks examined next have not yet incorporated the microbiome.

## 4 Current therapeutic drug monitoring and model-informed precision dosing: state of the art and the microbiome blind spot

TDM and MIPD have become a mature, covariate-driven discipline. They individualize therapy by combining measured concentrations with patient characteristics (Darwich et al., 2021). The sophistication of this apparatus makes one omission conspicuous: the covariate set these frameworks interrogate does not include the gut microbiome, despite the mechanistic and clinical evidence that microbial variation influences drug exposure. This section characterizes the current state of the art and locates that omission, because it is the omission, and not any deficiency in the underlying evidence, that this review sets out to correct.

The field has progressed from reactive concentration feedback toward predictive, model-based dosing. Classical TDM compares a measured concentration against a target range and adjusts empirically. MIPD instead embeds each measurement within a population pharmacokinetic model and uses Bayesian estimation to forecast the dose most likely to achieve a defined exposure (Mould and Upton, 2012). Vancomycin illustrates the transition. Consensus guidance now recommends area-under-the-curve, Bayesian-guided monitoring over trough-based dosing, because trough concentrations alone systematically misestimate exposure (Neely et al., 2014; Rybak et al., 2020). Structured model-informed approaches have been advocated for antibiotics more broadly (Wicha et al., 2021), and international guidance endorses concentration-guided individualization across several drug classes in the critically ill, where pharmacokinetics are most variable (Abdul-Aziz et al., 2020).

Supporting infrastructure has matured in parallel, with a growing ecosystem of dosing software. These tools remain unevenly integrated into practice, however, and depend heavily on the quality of the underlying model (Broeker et al., 2019; Kantasiripitak et al., 2020; Keizer et al., 2018). More recent appraisals confirm that the gap between methodological maturity and routine uptake has narrowed only partially (Kluwe et al., 2021; Pérez-Blanco and Lanao, 2022). The binding constraints they identify are implementation, workflow integration, and evidence of clinical benefit, not modeling capability. A focused systematic review of Bayesian MIPD in routine practice found adoption still concentrated in a few drugs, led by vancomycin, and in centres with local pharmacometric expertise (Alghamdi, 2026). Where implementation has been studied prospectively the limiting factors proved organizational: sampling logistics, clinician trust in model output, and the division of responsibility between pharmacist and prescriber (Swartling et al., 2025). This matters here because it shows the cost of adding a covariate to MIPD is procedural as well as statistical, a point taken up in Section 6.

The predictive power of these models rests on the covariates they encode. Population pharmacokinetic models routinely incorporate renal and hepatic function, body size, age, and drug interactions, and increasingly pharmacogenomic status, to explain systematic differences in clearance and volume of distribution. Consensus dosing frameworks for narrow-therapeutic-index agents such as tacrolimus specify these covariates explicitly (Brunet et al., 2019), and the “virtual twin” concept has clarified what patient data are needed to individualize a model reliably (Polasek and Rostami-Hodjegan, 2020). Machine-learning methods now extend the covariate space further. In a study of tacrolimus, machine-learning models trained on routinely collected data predicted exposure more accurately than the reference approach, indicating that additional, non-traditional variables can improve dose prediction when they carry real information (Woillard et al., 2021). This is a single study, in one drug, and its covariates were conventional clinical and demographic variables, not microbial ones. We cite it as evidence that the modeling architecture can absorb additional predictors, not as evidence that it can absorb microbiome features specifically. The architecture is therefore built to absorb new covariates, provided they are measurable and quantitatively linked to exposure.

Within this covariate-driven architecture, the gut microbiome is absent. None of the established population pharmacokinetic models, Bayesian dosing tools, or consensus monitoring guidelines treats microbial composition or enzymatic activity as an input, even for drugs whose disposition is demonstrably microbe-dependent. As a result, microbially driven variability, which the preceding sections show to be real and sometimes large, is not modeled as a structured covariate. It is instead absorbed into interindividual random effects and appears to the model as unexplained residual variability. Clinical pharmacology has begun to acknowledge the microbiome’s contribution to drug disposition and to pharmacokinetic-pharmacodynamic variability (Tsunoda et al., 2021), yet this acknowledgment has not been converted into a modeled input. The microbiome thus occupies an unusual position: a recognized determinant of exposure that sits entirely outside the very frameworks built to explain exposure. This is the blind spot that motivates the rest of the review.

Closing it does not require a new dosing paradigm. It requires adding one more covariate to an architecture already suited to receive it. The obstacle is translational: microbial contributions must be rendered as measurable, quantitatively defined variables that a population or physiologically based model can use. Proof that this is achievable already exists, in physiologically based pharmacokinetic models that represent microbial drug-metabolizing activity explicitly and predict its effect on systemic exposure (Zimmermann-Kogadeeva et al., 2020). That proof was obtained, as noted in Section 1, in gnotobiotic mice and not in patients. The next section builds on it to propose how the microbiome can be operationalized as a covariate within TDM and MIPD.

## 5 Integrating the gut microbiome into therapeutic drug monitoring and model-informed precision dosing

Correcting the blind spot identified above does not demand a new dosing paradigm; it demands that the microbiome be rendered in the same terms as any other covariate. We therefore propose an operational framework in which a microbial feature is measured, linked quantitatively to a pharmacokinetic parameter, and incorporated into a dosing model that already accepts covariates (Figure 2). Three requirements define the path: a microbial biomarker measurable in routine practice, a quantitative relationship between that biomarker and a defined pharmacokinetic or pharmacodynamic parameter, and a modeling route through which the biomarker adjusts the predicted dose. The following subsections address these requirements in turn (what to measure, how to sample and measure it, and how to model it), and then assemble them into the integrated workflow of Figure 2. Existing physiologically based models that represent microbial drug metabolism explicitly show that the endpoint is attainable (Zimmermann-Kogadeeva et al., 2020).

**FIGURE 2:**
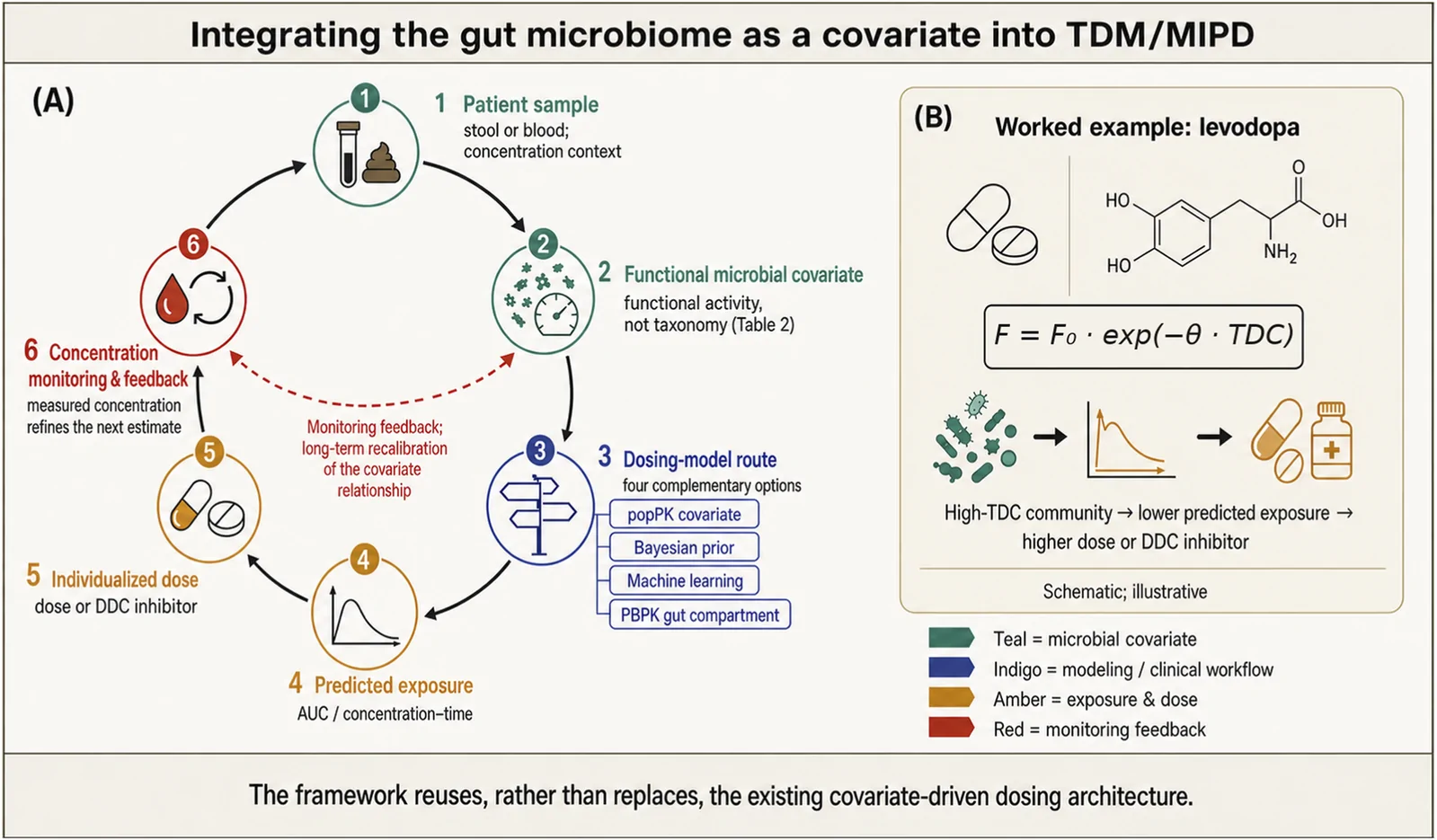
An operational framework for integrating the gut microbiome as a covariate into therapeutic drug monitoring and model-informed precision dosing. **(A)** A patient sample yields a functional microbial covariate (candidate biomarkers in Table 3), which enters one or more of four complementary modeling routes: as a covariate on a structural pharmacokinetic parameter within a population pharmacokinetic model, as a metabotype that selects or weights the Bayesian prior, as a feature within a machine-learning predictor of exposure, or as an intestinal enzymatic compartment within a physiologically based model. These generate a predicted exposure and an individualized dose, and subsequent concentration measurement feeds back both to refine the individual estimate and, over time, to recalibrate the covariate-exposure relationship itself. Data requirements, validation strategies, limitations, and prototyping status for each route are given in Table 1. **(B)** Worked example (levodopa): tyrosine-decarboxylase gene abundance (TDC) can be encoded as a covariate on bioavailability, F = F_0_·exp (−θ·TDC), so that a high-decarboxylase community is assigned a lower predicted exposure and, through Bayesian forecasting, a correspondingly higher dose or the addition of a decarboxylase inhibitor. This expression is hypothetical and illustrative only: θ has not been estimated from any dataset, no source data exist for it, and the exponential form is chosen for its mathematical convenience in constraining F to (0, F_0_] rather than because the underlying biology has been shown to follow it. A real model would additionally require gastric-emptying status, decarboxylase-inhibitor regimen, and dietary protein intake as competing covariates, and would have to be estimated on measured concentrations rather than dose requirements (Section 5.4.1). Two further worked examples of greater evidential maturity, irinotecan and mycophenolic acid, are developed in Sections 5.4.2 and 5.4.3. The framework reuses, rather than replaces, the existing covariate-driven dosing architecture.

### 5.1 What to measure: a functional microbial biomarker

The first requirement is a biomarker that reflects drug-relevant microbial function, not taxonomy alone. Community composition is an unreliable covariate, because taxonomically distinct microbiomes can share the same metabolic activity, and taxonomically similar ones can differ in the genes that matter for a given drug. Function-oriented biomarkers are preferable, and several candidates already have quantitative links to exposure. The abundance of bacterial tyrosine decarboxylase genes predicts the fraction of levodopa lost before absorption and correlates with dose requirement, making it a near-ready covariate for a specific drug (van Kessel et al., 2019). Measured gut β-glucuronidase activity indexes the reactivation of glucuronidated drugs, and therefore enterohepatic recirculation, defining a high-turnover metabotype relevant to agents such as irinotecan (Guthrie et al., 2017). Marker genes for defined metabolic transformations (illustrated by the *ismA* genes that predict a host metabolic phenotype) demonstrate the more general principle that a functional gene can serve as a quantitative covariate (Kenny et al., 2020). Broader functional readouts, including the fecal metabolome and the expressed metatranscriptome, capture aggregate activity that may be useful when no single enzyme dominates (Abu-Ali et al., 2018; Zierer et al., 2018). Table 3 summarizes these candidates and their measurement and modeling routes.

**TABLE 3:** Candidate gut-microbial biomarkers and their measurement and modeling routes.

Candidate biomarker	Measurement method	Linked PK/PD parameter	Modeling use	Practical readiness	Representative evidence
Tyrosine decarboxylase (*tdc*) gene abundance	qPCR/ shotgun metagenomics	Levodopa bioavailability (absorbed fraction)	Covariate on bioavailability (F)	Near-deployable: targeted qPCR on existing hospital platforms; low reagent cost; plausible turnaround <1 working day; assay not clinically standardized or validated	van Kessel et al. (2019)
Gut β-glucuronidase activity	Targeted enzyme-activity assay/ functional metagenomics	Enterohepatic recirculation; exposure & toxicity	Covariate on reabsorption/ apparent clearance	Near-deployable: direct activity readout; moderate cost; requires strict pre-analytical control because activity degrades; no standardized clinical assay format	Guthrie et al. (2017)
Community *ex vivo* drug-metabolizing capacity	*Ex vivo* drug-metabolism screen	Fraction metabolized (metabolizer phenotype)	Metabotype stratification of Bayesian prior	Research-grade: requires anaerobic culture and analytical chemistry; days of turnaround; not scalable to routine care	Javdan et al. (2020)
Functional marker gene (e.g., *ismA*, paradigm)	Metagenomic gene quantification	Gene-defined metabolic phenotype	Functional-gene covariate	Near-deployable in principle once the drug-relevant gene is identified (cited here as a paradigm for a different phenotype, not as a dosing biomarker)	Kenny et al. (2020)
Fecal metabolome	Untargeted metabolomics	Functional readout of microbial activity	Composite covariate/ model input	Research-grade: high per-sample cost, reference-laboratory dependence, cross-site comparability unresolved	Zierer et al. (2018)
Gut metatranscriptome	RNA-seq metatranscriptomics	Expressed (active) metabolizing capacity	Activity-weighted covariate	Research-grade: highest cost and technical demand; RNA lability makes ward-based collection impractical	Abu-Ali et al. (2018)

*F*, bioavailability; PK/PD, pharmacokinetic/pharmacodynamic; qPCR, quantitative polymerase chain reaction; *tdc*, tyrosine decarboxylase gene; *ismA*, intestinal steroid metabolism A gene. Gene names are italicized. Rows are ordered from the most nearly deployable single-gene covariates to aggregate functional readouts. Definition of readiness categories. “Near-deployable” means the measurement could in principle be performed on instrumentation already present in a hospital clinical laboratory, at a per-sample cost of the same order as existing therapeutic drug monitoring assays, with a turnaround compatible with dose adjustment during an admission (approximately one working day); it does not imply that a validated, standardized, or regulatorily qualified clinical assay exists, and for every biomarker listed it does not. “Research-grade” means the measurement currently requires specialist equipment, reference-laboratory referral, or bioinformatic support not generally available in hospital laboratories, with turnaround of days to weeks. No biomarker in this table has been analytically standardized for clinical use or qualified for any dosing context of use (Section 7).

Before any candidate is treated as a covariate, however, the confounding flagged at the end of Section 3 must be addressed at the level of biomarker definition, because it determines what a measured association can mean. Three consequences follow for biomarker selection. First, a biomarker specified as the abundance or activity of an enzyme with a known mechanistic role in the drug’s disposition is far more defensible than one from an untargeted association scan. The mechanistic link constrains the plausible confounders: tyrosine decarboxylase abundance is interpretable as a determinant of levodopa loss in a way that a taxon merely correlated with dose requirement is not. Second, any covariate model must carry the principal non-microbial determinants of the same endpoint as competing covariates. The microbial term should be estimated conditional on diet, disease severity, and co-medication, not allowed to stand in for them. Otherwise it will absorb variance it does not cause and fail on external validation. Third, the direction of causation cannot be assumed, since drugs reshape the microbiome as reliably as the microbiome reshapes drugs (Maier et al., 2018; Vich Vila et al., 2020). A cross-sectional association between a microbial feature and dose requirement is therefore compatible with the dose having produced the feature. For the biomarkers in Table 3 this is a design constraint and not a fatal objection. It is also the main reason the framework below requires prospective validation; retrospective validation will not settle the question.

### 5.2 How to sample and measure it: the pre-analytical and analytical layer

The second requirement is a measurement layer that yields these biomarkers reproducibly. In microbiome work the pre-analytical stage dominates the error budget, so specimen choice, collection, and storage deserve the same scrutiny a clinical laboratory would apply to a drug assay. We therefore treat sampling explicitly before turning to analytical platforms; Table 4 summarizes the options and their trade-offs.

**TABLE 4:** Microbiome sampling strategies for a dosing covariate: specimen, handling, timing, and trade-offs.

Dimension	Option	Strengths	Limitations	Recommended use for a dosing covariate
Specimen	Stool	Non-invasive, self-collected, repeatable, inexpensive; the only realistic routine option	Represents the distal colonic lumen only; under-represents the mucosa-associated community (Zmora et al., 2018); a surrogate for proximal small-intestinal metabolism rather than a measure of it	Default, and mechanistically appropriate for distal β-glucuronidase-dependent enterohepatic recirculation (irinotecan, mycophenolic acid)
	Small-intestinal contents (ingestible capsule device)	Samples the site of proximal metabolism; luminal microbial and bile-acid profiles differ substantially from stool (Shalon et al., 2023)	Cost and availability confine it to research; no clinical assay pathway	Validation phase for proximally metabolized drugs (levodopa); accept stool surrogates only once shown to track the proximal measurement
	Mucosal biopsy	Resolves the luminal-versus-mucosal discrepancy directly	Requires endoscopy; cannot support repeated or routine measurement	Not suitable for dosing; mechanistic studies only
Collection & storage	Immediate freezing (−80 ℃)	Reference condition; best preservation of nucleic acids and activity	Requires on-site freezer capacity; impracticable on many wards and in outpatient settings	Preferred where infrastructure exists
	Validated stabilizing buffer at ambient temperature	Feasible on a ward or at home; acceptable reproducibility when applied consistently (Costea et al., 2017; Vogtmann et al., 2017)	Buffer-specific bias; not interchangeable between buffers	Acceptable substitute, provided a single buffer is retained for all samples contributing to one model
	Enzyme-activity assays (any specimen)	Direct functional readout, closest to the pharmacokinetically relevant quantity	Activity, unlike DNA, degrades, so results are interpretable only against a documented handling protocol	Record and fix the collection-to-processing interval; treat it as a candidate covariate rather than assume it negligible
Frequency & timing	Single pre-dose sample	Lowest burden and cost	Day-to-day within-person variation is substantial against a stable personal baseline (Caporaso et al., 2011); may not represent the community present at dosing	Acceptable for a single acute decision; take the sample close to the dosing event
	Standardized time of day	Removes a genuine source of variation, since composition and function oscillate diurnally (Thaiss et al., 2014)	Constrains collection logistics	Required across all patients contributing to a model
	Repeated sampling during therapy	Tracks a moving covariate; the only defensible approach for therapy lasting weeks	Cost and patient burden; optimal interval unknown for every drug	Unresolved and drug-dependent; for chronic dosing (e.g., tacrolimus post-transplant) the assumption that one baseline sample remains representative is the least defensible element of the framework and an explicit target for prospective study
	Re-sampling after acute perturbation	Captures antibiotic-induced disruption, which is gradual, individual (Dethlefsen et al., 2008), and antibiotic-class-specific (Maier et al., 2021)	Adds unscheduled samples	Required after recent or concurrent antibiotics; do not rely on a pre-antibiotic value
Analytical platform	Targeted qPCR of one marker gene	Inexpensive, fast, deployable on existing hospital instrumentation	Measures only the designed target; gene abundance is not activity	Preferred for a first generation, since the framework needs one mechanistically specified activity per drug, not a community survey
	Shotgun metagenomics/ metatranscriptomics/ untargeted metabolomics	Hypothesis-free; captures aggregate function where no single enzyme dominates	Cost, reference-laboratory referral, days-to-weeks turnaround; inter-laboratory variation can exceed biological differences (Sinha et al., 2017)	Research and biomarker-discovery phase; not yet compatible with routine dosing economics (Section 7)
	Quantitative microbiome profiling (cell counts + sequencing)	Recovers absolute abundance, the quantity a PK model actually requires (Vandeputte et al., 2017)	Additional cell-counting step and cost	Prerequisite, not a refinement: relative abundance can rise while the absolute metabolizing population falls

PK, pharmacokinetic; qPCR, quantitative polymerase chain reaction. The overarching principle is that specimen choice should follow the mechanism and the anatomical site of metabolism, and that pre-analytical handling should be fixed and documented before any covariate is estimated, because inter-laboratory and pre-analytical variation currently exceeds the biological signal a dosing model would seek to exploit (Costea et al., 2017; Sinha et al., 2017).

#### 5.2.1 Which specimen

Stool is the only specimen that is non-invasive, self-collected, and repeatable, and is consequently the default for any covariate intended for routine use. Its principal limitation is that it represents the distal colonic lumen. For proximally metabolized drugs this is a genuine mismatch. Levodopa is decarboxylated in the proximal small intestine (van Kessel et al., 2019), a compartment stool represents only indirectly. The levodopa example in Section 5.4.1 must therefore be read as calibrated against a surrogate specimen, not the site of action. Stool also under-represents the mucosa-associated community, and mucosal and luminal profiles can diverge within the same individual (Zmora et al., 2018). Mucosal biopsy resolves that discrepancy but requires endoscopy, and so cannot support repeated measurement. Direct sampling of small-intestinal contents historically required intubation. Ingestible capsule devices now permit collection along the intestinal tract in ambulatory subjects, revealing luminal microbial and bile-acid profiles substantially different from stool (Shalon et al., 2023). This is the most mechanistically appropriate specimen for proximal metabolism, though it remains a research tool in cost and availability. The practical resolution is to make specimen choice follow the mechanism: stool for distal β-glucuronidase-dependent recirculation, capsule or aspirate sampling for proximal metabolism during validation, with stool surrogates accepted only once shown to track the proximal measurement.

#### 5.2.2 Collection and storage

Pre-analytical handling can introduce variation comparable in magnitude to the biological signal. Systematic comparison of fecal sampling and preservation protocols shows that collection method and storage condition measurably shift apparent community composition, and that protocol consistency matters more than any single protocol being optimal (Costea et al., 2017). Comparisons of collection devices in epidemiological settings reach the same conclusion (Vogtmann et al., 2017). Three operational implications follow for a dosing covariate. Immediate freezing at −80 ℃ remains the reference condition. Where that is impracticable on a ward, a validated stabilizing buffer should be used and retained for all samples contributing to a given model. The collection-to-processing interval should be recorded, not assumed negligible. Enzyme-activity assays impose a further constraint absent from sequencing: activity, unlike DNA, degrades, so such a measurement is interpretable only against a documented handling protocol.

#### 5.2.3 Frequency and timing

The microbiome is dynamic, so the sampling schedule is part of the biomarker definition and not a logistical detail. Dense longitudinal sampling shows substantial day-to-day variation within individuals against a stable personal baseline (Caporaso et al., 2011). Community composition and function also oscillate diurnally, making time of day a genuine source of variation (Thaiss et al., 2014). Three rules follow. The sample should be taken close to the dosing event whose exposure is predicted. Time of day should be standardized across patients contributing to a model. Acute perturbations require re-sampling, not reliance on a historical value. Recent or concurrent antibiotics are the clearest case: recovery after even a single course is gradual and individual (Dethlefsen et al., 2008), and disruption is class-specific (Maier et al., 2021). Whether a single pre-dose sample suffices, or the covariate must be updated during therapy, is drug-dependent and unresolved. For a drug dosed over weeks, such as tacrolimus after transplantation, the assumption that one baseline sample remains representative is the least defensible element of the framework, and an explicit target for prospective study.

#### 5.2.4 Analytical platform

The available methods trade depth against practicality. Shotgun metagenomics enumerates the genes present but not whether they are expressed. Metatranscriptomics captures expressed function but is more demanding and less stable (Abu-Ali et al., 2018). Targeted enzyme-activity assays report the specific activity of interest most directly, yet must be developed per pathway. Targeted quantitative PCR of a single marker gene sits at the opposite end of this trade-off from sequencing. It is inexpensive, fast, and already deployable on platforms present in hospital molecular laboratories, at the cost of measuring only what it was designed to measure. For a first generation of microbiome-informed dosing this limitation is acceptable, because the framework requires one mechanistically specified activity per drug and not a community-wide survey.

A further requirement is quantitation of microbial load, because relative-abundance profiling can misrepresent the absolute quantity of a metabolizing population. Quantitative microbiome profiling that combines cell counts with sequencing corrects this distortion (Vandeputte et al., 2017). This is a prerequisite and not a refinement: a pharmacokinetic model requires the absolute quantity of metabolizing capacity presented to the drug, and a relative abundance can rise while the absolute population falls. Whichever method is chosen, covariate-grade use will need standardized sampling and analysis, an expectation set by reference characterizations of the healthy microbiome and by the recognition that functional readouts such as the fecal metabolome must be measured comparably across sites (Human Microbiome Project Consortium, 2012; Zierer et al., 2018). The scale of that problem is documented. Multi-laboratory comparison by the Microbiome Quality Control project found inter-laboratory handling and bioinformatic choices drove more variation in community profiles than the biological differences between specimens (Sinha et al., 2017), and efforts to develop reference materials and reporting standards remain in progress (Amos et al., 2020). Until these are settled, a microbial covariate estimated in one centre cannot be assumed transferable to another. That constrains the multi-centre model development Section 5.3 requires, and bears directly on the regulatory question of Section 7.

### 5.3 How to model it: four covariate routes

The third requirement is a modeling route that converts the biomarker into a dose adjustment, and the covariate-driven architecture of MIPD offers four. Table 1 sets out, for each route, the data required, an appropriate validation strategy, the principal limitations, and whether any published prototype exists. The routes are ordered from empirical to mechanistic, and they differ less in ambition than in the evidence they presuppose. The text below does not repeat the table. It explains why each route’s evidential threshold sits where it does.

#### 5.3.1 Route 1: covariate on a structural pharmacokinetic parameter

The most direct route adds the microbial biomarker as a covariate on a structural pharmacokinetic parameter within a population pharmacokinetic model estimated by standard methods (Mould and Upton, 2012). The relevant parameter is bioavailability for a gut-metabolized drug, or apparent clearance for one subject to enterohepatic reactivation. What makes this route demanding is not its methodology, which is conventional, but the patient numbers it needs. That condition is easily underestimated. Both power and selection bias depend strongly on sample size, and stepwise selection in small datasets systematically overstates effect size (Ribbing and Jonsson, 2004). A microbial biomarker is also typically skewed, and its relationship to exposure is not known to be log-linear, so the functional form must be pre-specified and validated externally. Internal cross-validation will not do.

#### 5.3.2 Route 2: metabotype-informed Bayesian prior

A second route uses a microbial metabotype to select or weight the Bayesian prior. It exploits the finding that the choice of prior model materially affects predictive accuracy (Broeker et al., 2019), and aligns with the virtual-twin principle that individualization depends on the right patient data (Polasek and Rostami-Hodjegan, 2020). Its evidential threshold is markedly lower than Route 1’s, and the reason is statistical rather than conceptual: a dichotomous classification such as high-versus low-β-glucuronidase turnover needs far fewer patients to establish than a continuous covariate-parameter relationship. The *ex vivo* metabolizer phenotyping already demonstrated in humans supplies just this kind of classification (Guthrie et al., 2017; Javdan et al., 2020). This makes Route 2 the most workable near-term option. Its characteristic risk is also distinctive: a misclassified patient may be dosed on a systematically wrong prior, and so fare worse than under a pooled model.

#### 5.3.3 Route 3: microbiome features in machine-learning predictors

A third route incorporates microbiome features directly into machine-learning predictors of exposure, which suit high-dimensional inputs and have already improved dose prediction from routine data (Woillard et al., 2021). The proof of concept most often invoked is the prediction of postprandial glycemic response from microbiome plus host features (Zeevi et al., 2015). That study is genuinely informative about feasibility: a microbiome-inclusive model outperformed conventional predictors and was validated prospectively in an independent cohort. It must nonetheless be cited with precision. The predicted endpoint was a physiological response to food, not the systemic exposure of a drug, and the work lies outside pharmacokinetics. It establishes that microbiome features can carry non-redundant predictive information in a host-response model. It does not establish that they can predict drug exposure, and we are aware of no study that has tested the latter. Together with the single-drug, non-microbial character of the tacrolimus machine-learning evidence noted in Section 4, Route 3 rests on two studies from adjacent domains and on no direct demonstration. Its data requirement is the steepest of the four, because high-dimensional inputs with few patients is the classic setting for overfitting.

#### 5.3.4 Route 4: mechanistic representation in a physiologically based model

The fourth route represents microbial metabolism mechanistically, as an intestinal compartment with defined enzymatic activity within a physiologically based model. This approach has already been shown to predict systemic exposure as a function of microbial activity (Zimmermann-Kogadeeva et al., 2020). Its requirements are qualitatively different from those of the other three. It needs mechanistic parameters instead of a large patient cohort, which changes what kind of study would establish it. Where those parameters are available, the route can predict exposure in a patient never included in any training set. That is its key advantage, and it is the only one of the four that can extrapolate to an unstudied dose or formulation. The corresponding vulnerability is that predictions are most sensitive to the parameters that are least well characterized, particularly regional microbial load and transit. This is also the one route with an existing prototype: microbial drug-metabolizing activity has been represented explicitly and used to predict systemic exposure, including quantitative attribution of the microbial fraction of ΔAUC, in gnotobiotic mice (Zimmermann et al., 2019b; Zimmermann-Kogadeeva et al., 2020).

These routes are complementary rather than exclusive, spanning empirical to mechanistic modeling. It should be stated plainly, in answer to the obvious question, that only Route 4 has been prototyped, and only in gnotobiotic mice. To our knowledge Routes 1 to 3 have not been implemented for a microbial covariate even retrospectively or in simulation, and this review proposes rather than reports them. The nearest retrospective opportunities are nonetheless informative about where a prototype could begin. The Parkinson’s disease cohort relating tyrosine decarboxylase abundance to levodopa dose requirement (van Kessel et al., 2019) and the transplant cohort relating *Faecalibacterium prausnitzii* abundance to tacrolimus dose escalation (Lee et al., 2015) each pair a microbial measurement with a dosing endpoint. The prospective mycophenolic acid study pairs a microbial perturbation with measured human exposure (Dukaew et al., 2023). What none contains is the paired biomarker-and-concentration-time data Route 1 requires, since dose requirement and trough management are not substitutes for characterized exposure. The most economical first prototype would therefore be a Route 2 study. It would refit an existing population model for a microbially handled drug, with patients stratified by one pre-specified microbial activity, and compare predictive performance against the unstratified prior. This needs no new methodology and no mechanistic parameters, only one added measurement in a conventional pharmacokinetic study. We identify it as the concrete next step, not an accomplished result.

### 5.4 Worked examples

A framework of this kind is only as credible as the cases to which it can be applied, so we work through three, chosen to span the range of evidential maturity currently available rather than to imply uniform readiness. Levodopa is the most mechanistically direct but rests on associative human data; irinotecan has the clearest directly measurable enzymatic biomarker; mycophenolic acid has the strongest human pharmacokinetic evidence, including a prospective perturbation. None is ready for clinical implementation, and we state for each what is missing.

#### 5.4.1 Levodopa: a covariate on bioavailability

A concrete illustration clarifies how such a covariate would operate. Levodopa is a workable first case, because the abundance of the bacterial tyrosine decarboxylase gene predicts the fraction decarboxylated before absorption, a quantity that maps directly onto oral bioavailability. In a population pharmacokinetic model this relationship can be encoded as a covariate on bioavailability. As an illustrative form, F = F_0_ · exp (−θ · TDC), where TDC is the standardized decarboxylase abundance and θ its estimated effect. A patient with a high-decarboxylase community is then assigned a lower predicted exposure and, through Bayesian forecasting, a higher recommended dose or the addition of a decarboxylase inhibitor (Mould and Upton, 2012; van Kessel et al., 2019).

This expression requires an explicit caveat, because it is easy to mistake for an operational tool. It is hypothetical and illustrative only. The parameter θ has not been estimated from any dataset, and no source data are cited for it because none exist. The exponential form is chosen for a pragmatic reason: it constrains F to the interval (0, F_0_] and yields a log-linear covariate term of the kind population models conventionally accommodate. It is not chosen because the underlying biology has been shown to follow it. A saturable form would be at least as defensible mechanistically, since a decarboxylase population presented with a fixed oral dose can be saturated. Discriminating between these forms is an empirical question the available data cannot answer. The evidence actually supporting the relationship is an association between decarboxylase gene abundance and levodopa dose requirement (van Kessel et al., 2019). Dose requirement is a clinical endpoint reflecting both exposure and response, not a measured bioavailability. The sign and existence of the modeled effect on F are therefore inferred, not demonstrated, though both are mechanistically plausible.

A real model would also have to contend with three determinants of levodopa exposure that are at least as influential as microbial decarboxylation and are correlated with it. Gastric emptying is delayed in a large proportion of patients with Parkinson’s disease, and is itself a recognized source of erratic levodopa absorption (Marrinan et al., 2014). Delayed emptying prolongs proximal residence, so it plausibly increases microbial decarboxylation as well as altering absorption directly. The two effects are confounded, not additive. Co-administered aromatic L-amino acid decarboxylase inhibitors, carbidopa or benserazide, are given to essentially all patients, and the bacterial enzyme responds differently to them than the host enzyme does (Maini Rekdal et al., 2019; van Kessel et al., 2019). The covariate effect is therefore conditional on the inhibitor and its dose, and not a drug-independent property. Dietary protein competes with levodopa for the large neutral amino acid transporter and is a classical cause of within-patient fluctuation (Nutt et al., 1984). Diet is simultaneously among the strongest determinants of microbiome composition (Falony et al., 2016), which makes it a confounder of biomarker and endpoint alike. A credible levodopa model would therefore need to carry gastric-emptying status, decarboxylase-inhibitor regimen, and dietary protein as competing covariates, and be estimated on measured concentrations instead of dose requirements. We present the equation as a statement of the form such a covariate would take, and a target for the prototyping study of Section 5.3. It is not a dosing rule.

#### 5.4.2 Irinotecan: β-glucuronidase activity as a covariate on enterohepatic recirculation

Irinotecan offers the most directly measurable biomarker of the three, and illustrates enterohepatic recirculation rather than presystemic loss. The active metabolite SN-38 is glucuronidated by the host and excreted into the intestine. There bacterial β-glucuronidase deconjugates it back to the active form, prolonging exposure and driving the dose-limiting gastrointestinal toxicity. Inhibiting the bacterial enzyme alleviates that toxicity in animals (Wallace et al., 2010), and the structural diversity of these enzymes has been catalogued (Pollet et al., 2017). Critically, the relevant microbial property is an enzymatic activity measurable directly in stool, not a gene abundance proxying for activity. Human communities stratify reproducibly into high- and low-turnover metabotypes on this measurement (Guthrie et al., 2017).

The modeling route follows from the mechanism. Reactivation returns drug to the absorptive compartment, so activity acts on the apparent clearance of SN-38, or more mechanistically on a reabsorption term, and not on bioavailability. Under Route 1, measured activity enters as a continuous covariate on apparent clearance, requiring paired activity and SN-38 concentration-time data. Under Route 2, the high-versus low-turnover metabotype selects between two priors. That is the more realistic near-term design and the study we would propose first, since the classification already exists and needs only to be paired with conventional pharmacokinetic sampling. The clinical target differs instructively from levodopa. The object is not to raise the dose in a high-metabolizing patient. It is to identify, before the first cycle, the patient whose community will over-reactivate SN-38 and who therefore warrants closer toxicity monitoring or dose moderation. What is missing is equally clear: no study has related measured fecal β-glucuronidase activity to measured SN-38 exposure in patients, the assay has no standardized clinical form, and stool activity has not been shown to predict intestinal activity at the relevant site.

#### 5.4.3 Mycophenolic acid: the strongest current human evidence, and a drug-drug-microbiome interaction

Mycophenolic acid is the example with the strongest human pharmacokinetic support, and the only one of the three in which a deliberate perturbation of the microbiome has been followed by a measured change in human systemic exposure. The mechanism is the same reactivation chemistry as irinotecan. Mycophenolic acid glucuronide is excreted into the intestine and deconjugated by bacterial β-glucuronidase, returning active drug for reabsorption and producing the characteristic second peak in the concentration-time profile. Two lines of evidence bracket the effect. In mice, eliminating gut bacterial β-glucuronidase activity with oral vancomycin relieves mycophenolate-induced gastrointestinal toxicity, establishing the causal role of the microbial enzyme (Taylor et al., 2019). In humans, co-administration of trimethoprim-sulfamethoxazole alters the gut community and reduces systemic mycophenolic acid exposure (Dukaew et al., 2023). The human study is a Tier 2 observation in the grading of Section 3, and the closest existing analogue to the evidence a covariate would require.

For a clinical pharmacist this example is also the most recognizable. Mycophenolic acid is monitored, its enterohepatic recirculation is known to shape its concentration-time profile, and trimethoprim-sulfamethoxazole is co-prescribed routinely as *Pneumocystis* prophylaxis in the same transplant population that receives it. The interaction is therefore not hypothetical but a common co-prescription whose pharmacokinetic consequence is microbially mediated. Modeled through Route 1, β-glucuronidase activity would enter as a covariate on the reabsorption fraction or apparent clearance, with concurrent trimethoprim-sulfamethoxazole as an interacting covariate on the same term. Through Route 4, the same activity would parameterize deconjugation in an intestinal compartment. That is attractive because it would predict the altered profile shape and not only the altered total exposure. The gaps are specific: the vancomycin intervention is a mouse study untested in patients, the human interaction study measured community and exposure change but not β-glucuronidase activity as a quantified covariate, and neither reports the paired activity-and-exposure data a model would need. Mycophenolic acid nonetheless has the shortest path from current evidence to a testable prototype of any drug considered here, which is why we nominate it, with irinotecan, for the Route 2 study proposed in Section 5.3.

### 5.5 The integrated loop

Assembled, these components form the closed loop shown in Figure 2. A patient sample yields a functional microbial covariate, which enters one or more of the four modeling routes to generate a predicted exposure and an individualized dose. Subsequent concentration measurement feeds back to refine both the estimate and, over time, the covariate relationship itself. The loop is workable only if the covariate isolates the microbial contribution specifically, which is why the ability to separate host from microbial metabolism quantitatively is foundational to the framework rather than incidental to it (Zimmermann et al., 2019b). In this configuration the microbiome is no longer an unmodeled residual but an input handled by the same machinery that already accommodates renal function or genotype.

This reframing turns the blind spot of the previous section into a tractable extension of existing practice, but it does not by itself deliver a validated tool. The framework depends on biomarkers whose quantitative link to exposure has been established prospectively rather than by association, on measurement standardized to covariate-grade reproducibility, and on evidence that adding the microbial covariate improves dosing accuracy over models without it. That last criterion is the one by which any covariate earns inclusion. Clinical pharmacology increasingly recognizes the microbiome’s contribution to disposition and pharmacokinetic-pharmacodynamic variability (Tsunoda et al., 2021), and the framework proposed here specifies how that recognition can be operationalized. What it makes possible in day-to-day practice, and what still stands in the way, are the subjects of the next two sections.

## 6 Clinical pharmacy applications and actionable strategies

The framework of the preceding section can guide clinical-pharmacy practice before any of its modeling components is fully validated, because several of its implications are already actionable at the bedside. Four domains are immediately relevant to the pharmacist: managing the timing of drugs relative to antibiotic exposure, reviewing co-medications for microbiome-mediated interactions, stratifying and, where justified, intervening on high-risk patients, and educating patients and teams. These actions do not require a quantitative dosing model. They require recognizing when a drug is microbially handled and adjusting practice accordingly. Figure 3 organizes these judgments into a decision aid that begins from a single question, whether the drug in hand is subject to microbial metabolism or enterohepatic reactivation, and proceeds to concrete steps.

**FIGURE 3:**
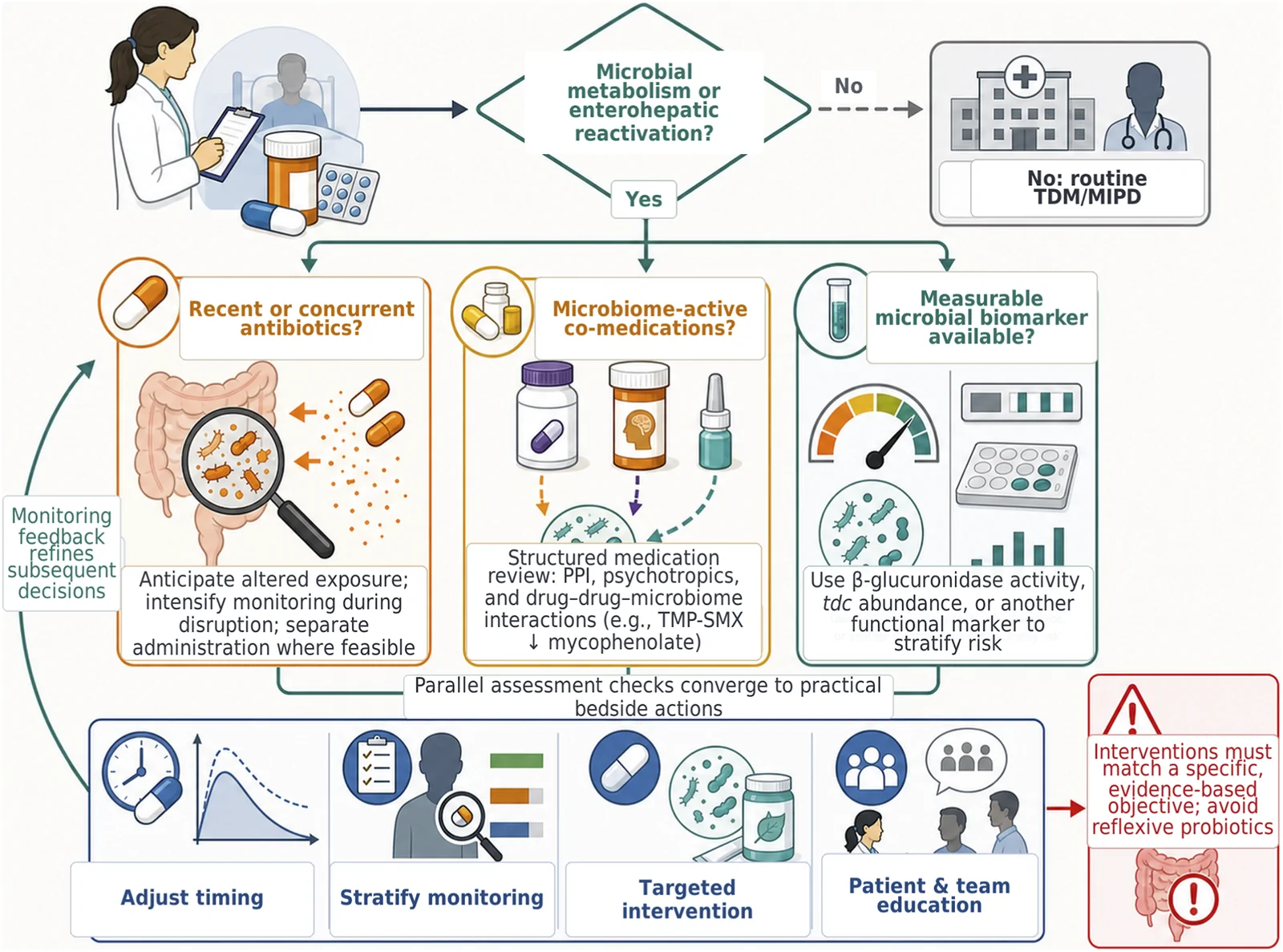
A decision aid for clinical pharmacists on microbiome-relevant dosing. Starting from whether a drug is subject to microbial metabolism or enterohepatic reactivation, the pharmacist assesses recent or concurrent antibiotic exposure (class-specific disruption of the drug-metabolizing community), microbiome-active co-medications (for example, proton-pump inhibitors, trimethoprim-sulfamethoxazole, and psychotropic agents), and any measurable microbial biomarker, and then acts by adjusting administration timing, stratifying monitoring, applying a targeted intervention, or educating the patient and care team. Interventions should be matched to a specific, evidence-based objective rather than applied reflexively, because microbiome manipulations such as empiric probiotics are not uniformly benign. Targeted interventions such as oral vancomycin to suppress bacterial β-glucuronidase activity should be read as mechanistic proof that the activity is manipulable, demonstrated in mice, and not as a clinical recommendation (Section 6). Monitoring feeds back to refine subsequent decisions. These actions are available now and require recognizing when a drug is microbially handled rather than a quantitative dosing model.

Antibiotic exposure is the most predictable perturbation of the drug-metabolizing microbiome, and therefore the clearest point of pharmacist intervention. Broad-spectrum antibiotics deplete a substantial fraction of gut taxa, and recovery after even a single course is gradual and individual, leaving a window during which microbial drug metabolism is altered (Dethlefsen et al., 2008). The effect is not uniform across agents. Systematic profiling of 144 antibiotics shows inhibition spectra that differ by class, together with species-specific killing within classes, so the expected disruption depends on the specific antibiotic chosen (Maier et al., 2021). Reconstitution of the community after antibiotics is itself modifiable and can be delayed by empiric probiotics, which complicates naive attempts to accelerate recovery (Suez et al., 2018). For a patient receiving a microbially metabolized drug alongside or shortly after antibiotics, the pharmacist can anticipate altered exposure, intensify monitoring during the disrupted interval, and consider separating administration in time where feasible.

Co-medications beyond antibiotics also reshape the microbiome or drive microbially mediated interactions, making structured medication review a natural extension of pharmacist practice. Screening of more than a thousand marketed drugs found that roughly a quarter of those with human targets inhibit at least one gut species, so microbiome-active co-medications are common and not exceptional (Maier et al., 2018). Population cohorts confirm that many widely used drugs measurably alter community composition and function (Vich Vila et al., 2020). Proton-pump inhibitors are a frequent example, producing consistent shifts in the gut community that can modify the metabolic background against which other drugs are handled (Imhann et al., 2016). The interaction can be specific and clinically consequential: co-administration of trimethoprim-sulfamethoxazole reduces systemic exposure to mycophenolic acid through microbial changes, a drug-drug-microbiome interaction that a concentration-guided pharmacist is positioned to detect (Dukaew et al., 2023). Psychotropic agents similarly interact bidirectionally with gut bacteria, warranting attention in polypharmacy (Cussotto et al., 2019). For this class the bioaccumulation of duloxetine by gut bacteria supplies a concrete mechanism (Klünemann et al., 2021). Flagging such co-medications during review identifies the patients in whom microbial contributions to exposure are most likely to matter.

Where a high-risk metabotype can be identified, stratification and targeted intervention become possible, although intervention must be undertaken cautiously. The high-turnover β-glucuronidase metabotype associated with irinotecan toxicity illustrates how a measurable microbial activity could triage patients toward closer monitoring or modified therapy (Guthrie et al., 2017). Targeted manipulation of a specific microbial activity is feasible in principle: eliminating gut bacterial β-glucuronidase activity with oral vancomycin relieves mycophenolate-induced gastrointestinal toxicity, a mechanistically rational intervention (Taylor et al., 2019). That demonstration was made in mice, however, and has not been replicated in patients. We cite it as mechanistic proof that the activity is a manipulable target, not as a basis for administering vancomycin to a transplant recipient. The antimicrobial-stewardship and colonization-resistance costs of doing so would be substantial, and have not been weighed against any demonstrated clinical benefit. More global manipulation through fecal microbiota transplantation is now governed by clinical-practice consensus and offers a defined, if substantial, means of altering the community (Cammarota et al., 2017). Caution is essential, however, because microbiome interventions are not uniformly benign. Empiric probiotics can impair rather than assist post-antibiotic recovery, so any intervention should be matched to a specific, evidenced objective and not applied by default (Suez et al., 2018).

A fair appraisal must also address what this would cost the pharmacist in time and workflow. A covariate that cannot be obtained inside the rhythm of clinical care will not be used, whatever its predictive value. The qualitative actions described above add little. Recognizing a microbially handled drug, checking recent antibiotic exposure, and flagging microbiome-active co-medications are extensions of medication review that draw on information already present in the record, and their marginal cost is measured in minutes.

The quantitative framework is different, and three burdens are worth naming. First, specimen logistics. Unlike a blood sample drawn on a scheduled round, a stool sample depends on patient defecation, cannot be timed precisely, requires patient instruction and willingness, and needs a ward collection and storage pathway that most units lack. Second, turnaround. A targeted quantitative PCR assay could plausibly return within a working day and inform a dose adjustment during admission. Metagenomic or metabolomic readouts return in days to weeks and could inform only subsequent cycles, so the framework is at present better suited to repeated or chronic dosing than to a single acute decision. Third, interpretation. A microbial covariate would arrive as a number requiring contextualization against assay-specific reference ranges, recent antibiotic exposure, and the confounders of Section 5.1. That is a new competency, not an extension of existing TDM interpretation, and it would have to be communicated to a prescriber with no prior framework for it. Experience with model-informed dosing is instructive here, because the barriers identified prospectively were organizational and not computational: sampling logistics, clinician trust in model output, and the division of responsibility between pharmacist and prescriber (Kluwe et al., 2021; Swartling et al., 2025). Microbiome-informed dosing would meet all three in sharper form. Our expectation, offered as a judgement and not a measurement, is that the incremental burden becomes justifiable only for narrow-therapeutic-index drugs in which microbial handling is mechanistically established and monitoring is already routine, a small set. First implementations should sit within research protocols where the sampling pathway is explicitly resourced.

These strategies are available now, yet they remain largely qualitative, and the clinical pharmacist occupies a dual position: applying them in practice while generating the prospective, concentration-linked data needed to make them quantitative. Proposals to harness the microbiome to improve drug therapy converge on just this translational role for pharmacy (Kelly, 2019), and moving microbiome-drug interaction research into routine care depends on systematic capture of exposure and outcome data at the point of dosing (Guthrie and Kelly, 2019). The immediate contribution of the pharmacist is therefore twofold: to act on the qualitative knowledge already available, and to document its effects rigorously enough to support the covariate-grade evidence the framework ultimately requires. The obstacles between this qualitative practice and quantitative, validated implementation are the subject of the final section.

## 7 Challenges and limitations

The framework proposed here is not yet a validated tool, and the obstacles that separate it from routine use fall into three levels: biological properties of the microbiome that complicate its use as a covariate, methodological limits in measurement and confounding control, and translational barriers of standardization, cost, and regulation. Delineating these honestly matters, because a microbial covariate must ultimately meet the same standard of validation as renal function or genotype before it can be trusted to guide a dose (Darwich et al., 2021). The challenges below can be overcome, but they define the distance still to cover.

The most fundamental difficulties are biological, because the microbiome is a moving target whose contribution is intertwined with other determinants of exposure. Dense time-series sampling shows pronounced variation in an individual’s microbiota across days and weeks, so a microbial covariate measured at one time may not represent the community present when a drug is dosed. Usable covariates must therefore be either temporally stable or measured close to the dosing event (Caporaso et al., 2011). Attribution is equally problematic. Microbial signatures are confounded with the disease being treated. The microbiome signature of type 2 diabetes, for instance, could only be resolved after controlling for antidiabetic medication (Forslund et al., 2015). Host and microbial pathways also frequently perform the same transformation, so the microbial fraction of variability can be isolated only through controlled measurement (Zimmermann et al., 2019b). As set out in Section 3, these are not deferred concerns but conditions on how the Tier 3 human evidence underpinning the framework should be read.

Methodological limits in measurement and confounding are more tractable but remain unresolved. Relative-abundance profiling can misrepresent the absolute size of a metabolizing population. Only quantitative approaches that incorporate microbial load recover the true quantity a pharmacokinetic model would need (Vandeputte et al., 2017). Population studies further show that a large share of microbiome variation is explained by diet, physiology, and concomitant medication, so any microbial covariate must be interpreted against a background of substantial, structured confounding (Falony et al., 2016; Zhernakova et al., 2016). Progress toward covariate-grade measurement will depend on standardized sampling and analysis benchmarked against reference characterizations of the healthy microbiome (Human Microbiome Project Consortium, 2012). Without that benchmark, results cannot be compared across sites or accumulated into the evidence base a dosing model requires. The size of the gap is quantified by the inter-laboratory variation documented in multi-centre comparison (Sinha et al., 2017), and it is only partly addressed by ongoing standards development (Amos et al., 2020).

The regulatory pathway deserves fuller treatment than it usually receives in this literature. It is the step at which a promising covariate either becomes usable or does not, and the requirements for a functional microbial biomarker differ in kind from those for the pharmacogenomic biomarkers the field is accustomed to. Formal qualification proceeds through a context-of-use statement that specifies the drug, the population, the decision the biomarker informs, and the consequence of being wrong. Analytical and clinical validation proportionate to that consequence then follow (Amur et al., 2015).

Four features of a microbial biomarker make this harder than for a germline genotype. First, the measurand must be defined operationally and not by identity. “Gut β-glucuronidase activity” is not a molecule but an aggregate catalytic capacity contributed by structurally diverse enzymes across many taxa (Pollet et al., 2017), so qualification would require agreement on substrate, assay condition, and unit before analytical validation could begin. Second, analytical validation needs reference materials and inter-laboratory reproducibility data the field does not yet possess, just the deficit multi-laboratory comparison exposed and standards efforts are attempting to close (Amos et al., 2020; Sinha et al., 2017). A genotype assay, by contrast, can be validated against a known sequence. Third, and most consequentially, the analyte is not stable. A germline variant is measured once and remains valid for life, whereas a microbial activity changes with diet, disease, and antibiotic exposure. Qualification must therefore specify the validity interval of a measurement and the circumstances requiring re-measurement, a dimension with little precedent among clinical biomarkers. Fourth, clinical validation would have to demonstrate not correlation with exposure but improvement in dosing accuracy or outcome over the same model without the biomarker. That is the standard MIPD applies to any candidate covariate (Darwich et al., 2021), and no microbial biomarker has been tested against it. A realistic sequence therefore runs from a single mechanistically specified activity in one drug and population, through an analytically standardized assay with published reproducibility, to a prospective study showing improved exposure prediction, and only then to a qualification submission. A plausible interim route is a laboratory-developed test at a single accredited centre, permitting prospective data generation without requiring qualification first. So that the ambition is not overstated: no microbial biomarker has to our knowledge entered a formal qualification programme for any dosing-related context of use.

Cost and cost-effectiveness form a further barrier that the literature, including our own earlier framing, has tended to leave implicit. The relevant comparison is against the assay the proposal would supplement. Routine immunoassay or chromatographic quantification of a drug concentration is inexpensive, high-throughput, and already reimbursed. A targeted quantitative PCR measurement of a single marker gene is of broadly comparable order. It is the only option in the framework that could plausibly be added without changing the economics of monitoring, which is a further argument for beginning with single-activity biomarkers and not community-wide profiling. Shotgun metagenomics, metatranscriptomics, and untargeted metabolomics are substantially more expensive per sample, require capital equipment and bioinformatic staff most hospital laboratories lack, and would be sent to reference laboratories, adding cost and turnaround. To this must be added costs easily omitted from an assay-level comparison: the specimen-collection pathway of Section 6, storage capacity, quality control, and pharmacist interpretation time.

Against these sits the potential benefit, avoided toxicity or therapeutic failure in drugs where both are expensive. The economic case is therefore most plausible where the clinical case is, namely, narrow-therapeutic-index drugs with costly failure modes such as immunosuppressants and cytotoxics. We are aware of no cost-effectiveness analysis of microbiome-informed dosing, and it would be premature to attempt one. The central input, the incremental gain in dosing accuracy attributable to the microbial covariate, has not been measured for any drug. What can be said is that cost-effectiveness should be a design constraint from the outset, not a downstream question. A framework requiring metagenomic sequencing for every dose adjustment will not be adopted, whereas one requiring a single inexpensive targeted assay in a selected population might be. This should govern biomarker selection as much as predictive performance does.

Translational barriers beyond cost and regulation are the least explored and may prove decisive. No microbial biomarker assay currently combines the turnaround time, cost, and regulatory qualification expected of a clinical covariate measurement. Nor have the prospective studies been performed that would show a microbial covariate improves dosing accuracy, rather than merely correlating with exposure. This is the same evidentiary bar that MIPD applies to any candidate covariate before adoption (Darwich et al., 2021; Tsunoda et al., 2021). It bears repeating in closing that, as Section 5.3 states, three of the four modeling routes proposed here have no published prototype of any kind, and the fourth has been prototyped only in gnotobiotic mice. The implication is not that the framework is impractical, but that its realization is a research and implementation agenda, not a finished capability. The concrete steps along that agenda, and the trajectory from the current concept toward microbiome-informed dosing in practice, are set out in the concluding section.

## 8 Conclusion and future directions

This review has argued that the gut microbiome should be treated not as a mechanistic curiosity but as a measurable, dosing-relevant covariate, to be integrated into therapeutic drug monitoring and model-informed precision dosing. Reframed this way, the microbiome ceases to be an unexplained source of residual variability and becomes an input that the existing covariate-driven dosing architecture is already equipped to accept.

The value of this reframing is concrete. A functional microbial covariate can help explain interindividual differences in exposure that pharmacogenomics leaves unaccounted for. It can support metabotype-based prediction of drug exposure and response. And it can inform practical decisions available to clinical pharmacists today: the timing of drugs around antibiotic exposure, the review of microbiome-active co-medications, and the stratification of patients toward closer monitoring or targeted intervention.

Realizing this value depends on progress across connected fronts. Experimental models that combine gnotobiotics with bacterial genetics should keep attributing variability, in quantitative terms, to host versus microbial processes. Functional microbial features need to enter population-pharmacokinetic and Bayesian dosing models as covariates. Measurement must advance toward quantitative, standardized readouts suitable for routine use. And machine-learning methods can be harnessed to exploit the high-dimensional structure of microbiome data within dosing prediction. If a single next step were to be named, it would be the study specified in Section 5.3: add one pre-specified microbial activity measurement to a conventional pharmacokinetic study of a microbially handled, routinely monitored drug, stratify an existing population model by that measurement, and test whether predictive performance improves. Mycophenolic acid and irinotecan are the best-positioned candidates. Such a study requires no new modeling methodology, and until one is done the framework proposed here remains a specification awaiting its first test.

These fronts form a closed loop, not a linear checklist. Solving the methodological and translational challenges will enable the clinical applications, and deploying the framework in practice will in turn generate the prospective, covariate-grade evidence that refines, validates, and where necessary corrects the underlying concept. Advancing along this loop is what will move the field from recognizing that gut microbes shape drug response toward microbiome-informed precision dosing at the bedside: an evolution from knowledge of microbial drug metabolism into a routine dimension of individualized therapy.
